# Total pelvic exenteration for pelvic recurrence after advanced epithelial ovarian cancer – A case report and literature review

**Published:** 2015

**Authors:** N Bacalbașa, I Bălescu

**Affiliations:** * “Carol Davila” University of Medicine and Pharmacy, Bucharest, Romania; ** “Ponderas” Hospital, Bucharest, Romania

**Keywords:** advanced stage ovarian cancer, pelvic recurrence, total exenteration

## Abstract

Ovarian cancer is an aggressive disease, which, although associated with a high rate of recurrence, seems to benefit most from iterative cytoreduction. Although the main patterns of its spreading are represented by peritoneal, lymphatic or hematogenous route, local recurrences might also be seen. Whenever pelvic recurrence develops, complete resections based on the ultraradical principles applied in resections for pelvic recurrences originating from cervical cancer, are useful. We present the case of a 56-year-old patient who was diagnosed with a pelvic recurrence invading both the urinary bladder and the anterior rectal wall two years and a half after a surgery for stage IIIC ovarian cancer.

## Introduction

Ovarian cancer is an aggressive disease with high capacity of developing recurrences, the main patterns of its spreading being represented by hematogenous, lymphatic and peritoneal route [**1**]. Although not so frequent, isolated pelvic recurrences might also be seen; in these cases, the principles of ultraradical pelvic surgery, which were first implemented in recurrent cervical cancer, seem to be successfully applied [**2**]. 

## Case report

**Fig. 1:**
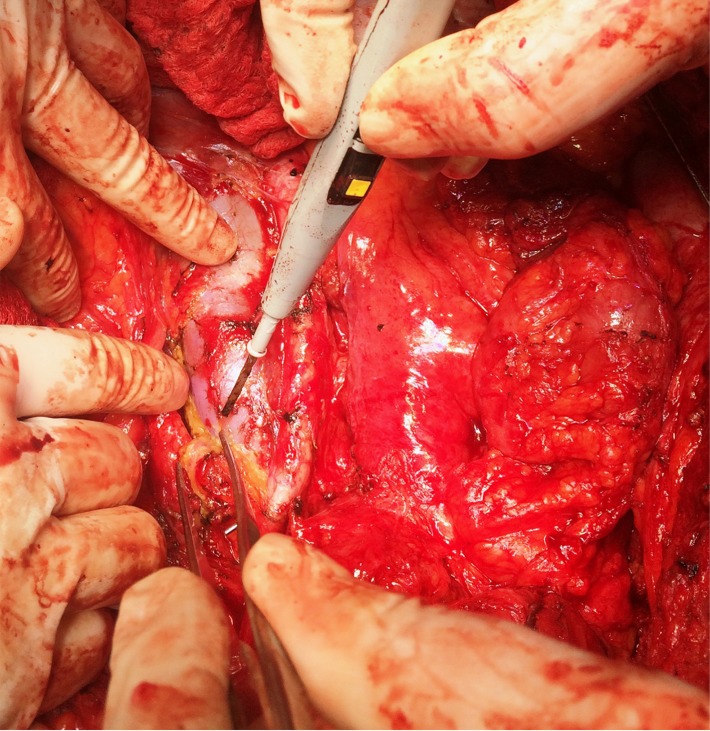
Para-aortic lymph node dissection

**Fig. 2:**
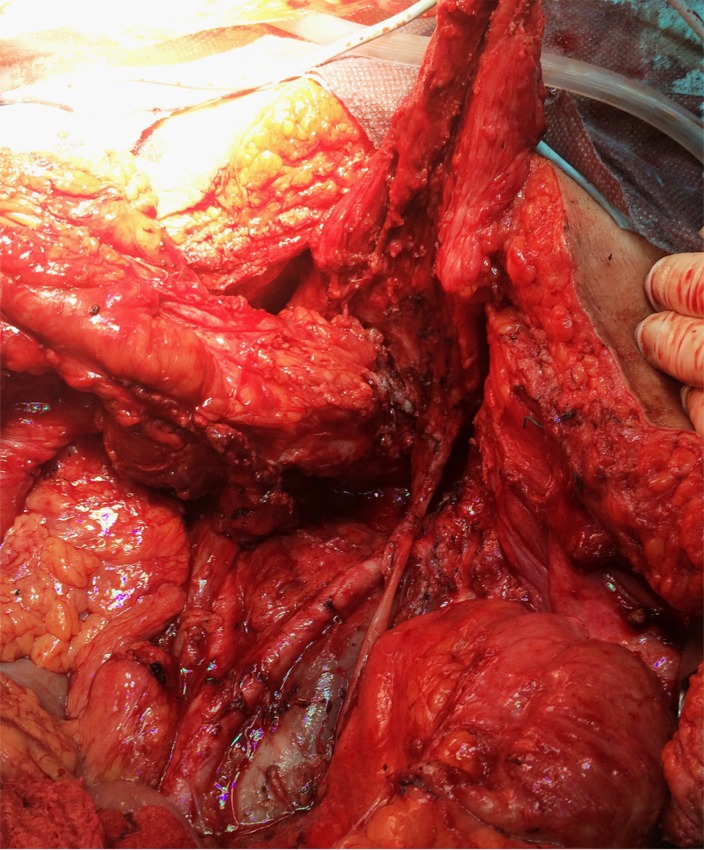
Large pelvic recurrence invading the rectum and the urinary bladder

**Fig. 3:**
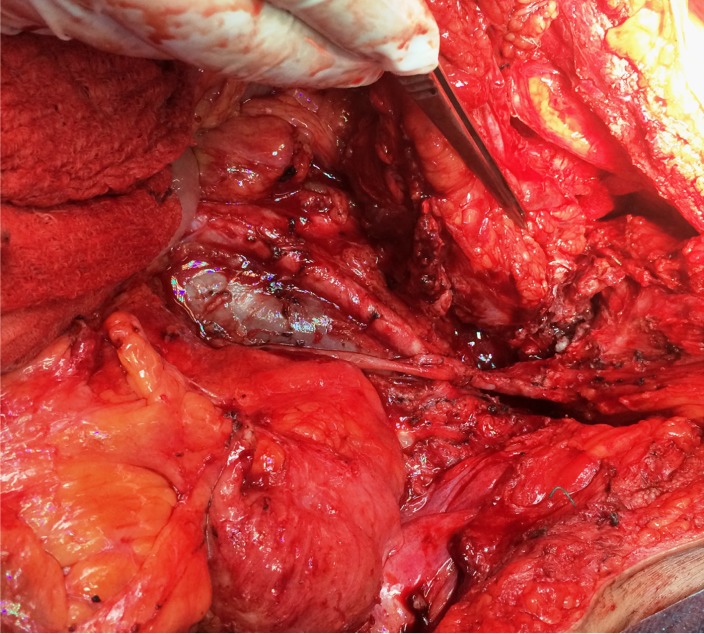
The final aspect after tumor mobilization

**Fig. 4:**
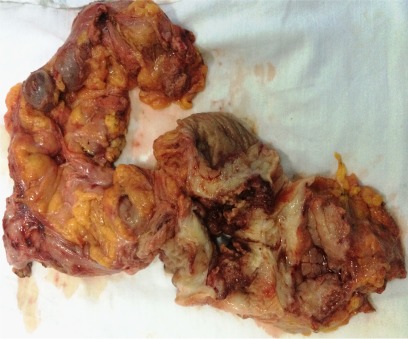
The specimen: recurrent ovarian tumor with rectal and urinary bladder invasion

The case of a 56-year-old patient, who was previously diagnosed with stage IIIC ovarian cancer, is presented. At that moment, a total hysterectomy with bilateral adnexectomy, omentectomy, parietal and pelvic peritonectomy were performed. Postoperatively, the patient underwent six cycles of adjuvant chemotherapy based on platinum salts and taxanes. Two years and a half after the completion of chemotherapy, the patient was investigated for pelvic pain and constipation; she was diagnosed with a pelvic recurrence invading both the urinary bladder and the anterior wall of the rectum. A total exenteration with pelvic and para-aortic lymph node dissection was performed (**Fig. 1**-**3**); the two ureters were exteriorized by end right sided cutaneous ureterostomy while the left colon was exteriorized in an end left sided colostomy. The histopathological examination of the specimen (**Fig. 4**) revealed the presence of a moderately differentiated serous epithelial ovarian cancer. The patient was discharged in the 11th postoperative day; at one year follow up, the patient was free of any recurrent disease.

## Discussions

Solitary pelvic recurrence after advanced stage epithelial ovarian cancer is not a very frequent condition [**2**]. However, experience achieved in treating local pelvic recurrences after cervical cancer was successfully implemented in treating pelvic recurrences originating from other tumors such as uterine cancer, bladder cancer, rectal cancer or even ovarian cancer [**3**]. Although initially when Brunschwig introduced it for the first time, pelvic exenteration was associated with unacceptable rates of postoperative morbidity and mortality, in time, this procedure became the corner stone in treating advanced pelvic malignancies [**4**,**5**]. At that moment, Brunchwig and Barber also reported a series of 22 patients submitted to pelvic exenteration for primary and recurrent ovarian tumors [**6**]. A few years later, Hudson and Chir described a radical pelvic approach involving radical oophorectomy associated with various resections of colic segments in order to provide complete macroscopic resection in primary advanced ovarian cancer [**7**,**8**]. Associated procedures employed to achieve complete cytoreduction included reverse hysterocolposigmoidectomy, modified posterior exenteration or total exenteration; however, they referred especially to colic resection during primary cytoreductive surgery [**9**-**11**].

When it comes to advanced ovarian cancer, an important matter of debate is the recurrent character of the disease which is responsible for the high number of relapses; this fact enables us to consider it rather a chronic disease characterized by asymptomatic periods (quantified as disease free survival) followed by symptomatic moments when recurrences are diagnosed. For cases initially diagnosed with advanced stage ovarian cancer, recurrence is expected to occur in 50 up to 90% of the cases, depending on individual risk factors and the type of associated oncologic treatment. It has been demonstrated that almost half of the patients diagnosed with recurrent ovarian tumors will associate a pelvic recurrence while almost 22% of the cases diagnosed with recurrent tumors will develop a solitary pelvic recurrence [**12**].

When it comes to pelvic recurrences after ovarian cancer necessitating pelvic exenteration, this is a quite rare condition. In their study, Wilt et al. included 42 patients diagnosed with advanced pelvic malignancies submitted to pelvic exenteration; however, a single patient was initially diagnosed with advanced stage epithelial ovarian cancer [**2**].

In another study conducted by Maggioni et al. involving 106 patients submitted to pelvic exenteration, only four of them reported pelvic recurrences originating from ovarian tumors [**13**].

Bristow et al. conducted a study on 56 patients submitted to secondary cytoreductive surgery in whom recto-sigmoidian resections were associated; in eight cases, urinary bladder or distal ureter invasion were also performed. The high incidence of pelvic recurrences involving both the rectal wall and the posterior wall of the urinary bladder were related to the local modifications of the pelvic anatomy after performing a radical hysterectomy with bilateral adnexectomy. Once the uterus and the cervix were removed, the anatomical relationships between the remnant organs changed, with a posterior displacement of the urinary bladder and an anteriorization of the rectum, which would partially cover the vaginal stump. In time, once a pelvic recurrence developed, a local invasion of the remnant pelvic viscera would produce much more easily when compared to cases which had not been submitted to previous surgical procedures and in which the natural compartmental barriers still existed. In Bristow’s study, the urinary resection resumed to partial cystectomy or distal ureterectomy; secondarily, the continuity of the urinary tract was re-established by ureteroneocystostomy and psoas hitch, with or without a Boari flap. When studying the postoperative complications, the same study reported an incidence of 5,4% for bowel fistulas or pelvic abscess and 3,6% for postoperative pyelonephritis. Other encountered complications were not directly related to the extended pelvic resections and included wound infections, pulmonary embolism, pneumonia or adult respiratory distress syndrome. Early postoperative death was encountered in a single case and was related to a colic fistula, which necessitated early reoperation; however, death occurred due to septic syndrome [**14**]. 

In the study conducted by Nguyen et al., 76 patients with advanced pelvic malignancies were submitted to pelvic exenterations, 16 of them being diagnosed with ovarian cancer. At the moment of performing the exenteration, 5 cases were diagnosed with primary advanced stage ovarian cancer while the other 11 cases were diagnosed with pelvic recurrences after advanced ovarian cancer. The main surgical procedures performed involved anterior exenteration (4 cases with primary tumors and 3 cases with pelvic recurrences), posterior exenteration (in all cases posterior exenteration was performed for pelvic recurrences) and total pelvic exenterations (one case for primary ovarian cancer and 4 cases for pelvic recurrences). In two cases in which total exenteration was performed, radical resection was impossible to achieve; in all the other cases, a complete R0 resection was performed. No postoperative death was reported [**15**].

## Conclusions

Although it is not a very frequent condition, isolated pelvic recurrence after advanced stage ovarian cancer might be encountered; once a positive diagnosis is performed an aggressive surgical approach in order to completely remove the tumor is justified. At this moment, a compilation between the principles of ultraradical surgery implemented in advanced stage cervical cancer and the principles of maximal cytoreductive surgery, which were first postulated by Meigs and later demonstrated by Grifiths, seem to offer the best therapeutical option in order to provide an adequate local control of the disease. In the same time, the experience accumulated in ultraradical pelvic surgery for advanced stage or recurrent cervical cancer offers the surgeon the possibility to perform a complete resection with minimum postoperative complications and to offer the patient a maximal benefit in terms of survival.
